# A study on synergistic effect of chloride and sulfate ions on copper corrosion by using electrochemical noise in asymmetric cells

**DOI:** 10.1038/s41598-022-18317-2

**Published:** 2022-08-23

**Authors:** Ghazal Sadat Sajadi, Vahid Saheb, Mehdi Shahidi-Zandi, Seyed Mohammad Ali Hosseini

**Affiliations:** 1 Department of Chemistry, Shahid Bahonar University of Kerman, Kerman, 76175 Iran; 2 Department of Chemistry, Kerman Branch, Islamic Azad University, Kerman, Iran

**Keywords:** Chemistry, Engineering, Materials science

## Abstract

The current study includes a systematic examination of copper corrosion initially in each of NaCl and Na_2_SO_4_ solutions separately and then in the mixture solution of Cl^−^ and SO_4_^2−^ ions as aggressive ions. Electrochemical current noise (ECN) signals resulting from asymmetric (Asy) as well as symmetric (Sym) cells have been interpreted using wavelet transform (WT) along with statistical procedures. The signals have been detrended and the decomposition of every signal has been carried out into 8 crystals. Then the standard deviation of every crystal has been illustrated with the standard deviation of partial signal (SDPS) plots. The Asy electrodes increased the pitting detection on copper compared with the Sym ones, indicating higher efficiency of the Asy electrodes. Asymmetric-copper electrodes have been studied using SDPS plots at different temperatures (40, 60, as well as 80 °C). Finally, in order to partly understand the effect of Cl^−^ and SO_4_^2−^ ions on the corrosion of copper, the stabilization of Cu^2+^ cations by Cl^−^ and SO_4_^2−^ ions in aqueous solutions have been modeled by DFT calculations. The derived results are in accordance with the experimental data.

## Introduction

Corrosion includes natural processes through which the electrochemical oxidization of metals takes place to different compounds such as ions and oxidants^1^. The corrosion resistance of some materials is also reduced by several corrosive ions^2^. Pitting corrosion is a type of localized corrosion generating tiny holes on the surface of the metals, which may occur because of aggressive ions including chloride, sulfate and nitrate ions, and so on^3^. Different industries use copper and its alloys since they have great mechanical as well as thermal features. Electronics, plumbing, transmission wires, and heat exchangers are examples of the areas in which these metals are used^4–6^. A layer of oxide formed on the surface of copper in the presence of oxygen shows resistance to corrosion^7–9^. Nevertheless, restrictions in protecting and susceptibility to various corrosions, including uniform and pitting under specific corrosive species are some problems with copper oxide^10–12^. Besides, the copper corrosion procedure is accompanied by thermal activation in which copper dissolution kinetic increases due to increased temperature, and copper oxide stability is dependent on the temperature variations^13^.

Electrochemical noise (EN) applications in industries, particularly in dealing with remote corrosion as well as the online examination of corrosion type, are an interesting topic among researchers and scholars from different fields^14–18^. There is considerable destruction in pitting corrosion, which is a quick and sudden event. It is also difficult to measure these types of corrosion. Corrosion is charge generating which causes natural potential and current transients, that EN measurements can recognize such impulsive transients, called signals^16,19^. EN analysis is carried out through two major approaches, including the statistical and spectral techniques. Several parameters such as standard deviation, skewness, and kurtosis can be computed by statistical analyses. The first one indicates the electrochemical activities on the surface of electrodes. The second one measures symmetry about the mean, and the last one shows the peaked or flat distribution. The statistical approach requires pretreatments before analyzing data, because of the DC drift and non-stationary state^20–27^. In ideal conditions, identifying the type and intensity of the corrosion should be performed using data analysis procedures that do not need stationary or linear conditions while showing a high potential to distinguish in time and frequency domains at the same time. In this regard, novel multi-resolution time and frequency analyses, according to the wavelets, are good alternatives to deal with the existing challenges efficiently. Wavelets form the principal component of decomposing signals in wavelet transform (WT), which act similar to goniometric functions with various frequencies in Fourier Transform^28–32^.

The results obtained from the wavelet analysis process are shown by the graph of partial signal (PS) versus time, the energy distribution (ED) versus crystal number as well as the standard deviation of partial signal (SDPS) against crystal. The corrosion timescale and frequency range along with the associations with the number of crystals are shown in Table 1. Accordingly, as the number of crystals increases, the timescale and frequency range of corrosion also increase. The WT and SDPS curves have been frequently reported^33–35^. Information provided by the SDPS plot is more worth compared to the ED plot information since the value of the SDPS is associated with oscillations number and amplitude of signals, making it a good choice for the present study^35^.

**Table 1:** Frequency and timescale ranges for sampling frequency 4 Hz.

Crystal name	Frequency range/Hz	Timescale range/s
d1	4–2	0.25–0.5
d2	2–1	0.5–1
d3	1–0.5	1–2
d4	0.5–0.25	2–4
d5	0.25–0.125	4–8
d6	0.125–0.0625	8–16
d7	0.0625–0.0312	16–32
d8	0.0312–0.0156	32–64

A cell having two similar working electrodes (WEs) was called symmetrical (Sym) cell, with identical materials, sizes and surface preparation, these WEs connected with a zero-resistance ammeter (ZRA) coupling current between the electrodes^36^. Recent evidences have revealed that asymmetrical (Asy) cells having WEs with quite different sizes improve noise detection because the phenomena on one electrode are recorded^27,33,34,37^. These cells are capable of keeping time width in the SDPS plot, increasing the EN current transient amplitude, and preventing the recording of excess signals produced in Sym cells.

In the present study, in addition to EN measurements, the pitting corrosion behavior of pure copper is explored by electrochemical impedance spectroscopy (EIS) analyzing the corrosion resistance and polarization method quantifying the over potential (η)^38–43^. The synergistic effect of Cl^−^ and SO_4_^2−^ ion for corroding copper metal have also studied. As a consequence, experiments have been performed initially in the NaCl and Na_2_SO_4_ solutions separately and then in the mixture solution of Cl^−^ and SO_4_^2−^ ions (at 60 °C). To the best of our knowledge, the present work is the first report on studying pitting corrosion of copper by ECN signals using Asy copper electrodes. The current research aimed at showing that this type of configuration using copper could be a promising alternative to process ECN signals. Analysis of the results of Asy cells was followed by their comparison with Sym cells through the statistical and WT approaches. In addition, density functional theory (DFT) calculations are also undertaken to simulate the stabilization process of Cu^2+^ cations in aqueous media. These calculations reveal partly how Cl^−^ and SO_4_^2−^ ions facilitate the corrosion process of copper metal.

## Materials and methodology

### Experimental details

Preparation of three various sizes of copper specimens (rod type) took place, including symmetric in large (314–314 mm^2^) and small (3.14–3.14 mm^2^) sizes, and asymmetric cell (3.14–314 mm^2^) denoted as Sym-L, Sym-S and Asy, respectively. Soldering of the electrodes to a copper wire was performed after which they were completely covered with resin in a way that one surface, with the specified area, was exposed to the solution as the WE surface.

Silicon carbide papers (at a range of 400#–2000#) were used to abrade WE, after which distilled water was used for cleaning. Acetone was applied for degreasing before each running. The edges of sample were coated by nail polish to prohibit the crevice corrosion.

The experiment cells included at first 0.6 M Na_2_SO_4_ solution, then 0.6 M NaCl solution at 60 °c and finally 0.3 M NaCl + 0.3 M Na_2_SO_4_ solution at various temperature 40, 60 and 80 °C. The pH of all solutions was adjusted to 6 using a pH meter by drop wise addition of 1 M HCl or 1 M NaOH. The required salts were provided by Merck Company. No additional purifications were performed on the reagents.

Two face-to-face copper electrodes were located in the solution with a gap of 1 cm to form the electrochemical cell. Autolab 302 N potentiostat was used to monitor the ECN signals, EIS and polarization measurements. Recordings of ECN signals were performed following two hours of exposure of WEs in the solution for a 900-s interval. Application of the orthogonal Daubechies wavelets of the fourth order (db4) helped to perform the wavelet technique and the frequency of 4 Hz was used for the sampling frequency of the ECN data. Calculation of statistical parameters is carried out following the wavelet trend removal procedure. Three-electrode system including Ag/AgCl electrode, platinum plates, and copper as a reference electrode, a counter electrode, and the working electrode, respectively, was used to perform the EIS and polarization tests. The EIS measurements were carried out at open-circuit potential in the frequency range 100–1 × 10^–5^ kHz, applying a 10 mV sinusoidal perturbation. The polarization scan rate of 1 mV/s was considered for polarization curve plotting, prepared through automatic changes in the electrode potential at a range of open circuit potential toward negative potential (about − 0.6 V). *Matlab* software was used to perform the processing data of ECN and *Nova* softwares was utilized to analysis EIS and polarization curves. A number of 3 repetitions were considered for the experiments.

### Theoretical details

The geometries of all molecules in the reaction were optimized by B3LYP-DFT method employing 6–311++G (2d, p) basis set^39^. The effects of remaining water solvent molecules are implicitly accounted for and supposed to occur in a media with a dielectric constant of 78 (that for water). A continuous polarizable continuum model (CPCM) was employed to calculate the energies of the molecules that appeared in the reactions^40^. The DFT calculations were executed by Gaussian 09 program.

## Results and discussion

### EN measurement

**Figure 1:**
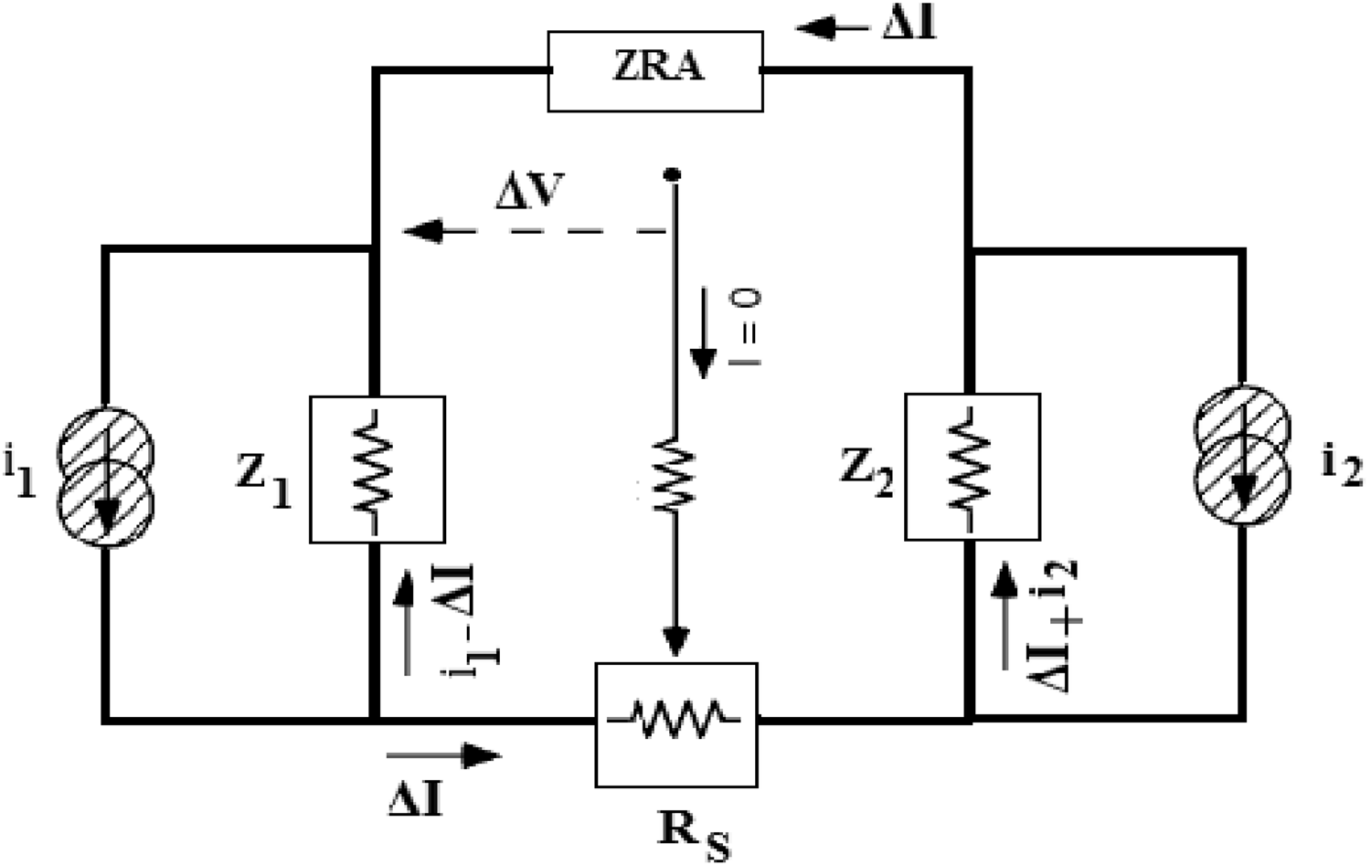
The equivalent circuit of corrosion cell to measure EN.

Figure 1 shows the equivalent circuit of corrosion cells to measure ECN, in which i_1_ and i_2_ are the sources of current noise originated from a localized phenomenon, for example evaluation and separation of bubble and corrosion in particular location. These are not accessible directly to measure, but the flowing current between two electrodes (ΔI) is measurable. The depiction of dynamic action of corrosion cells is the purpose of the equivalent circuit. Accordingly, it uses for all cells including two WEs. Utilizing Ohm’s law in the equivalent circuit, one can write, in the frequency domain^41^:

1 $$\Delta I=\frac{{Z}_{1}{i}_{1}-{Z}_{2 }{i}_{2}}{{Z}_{1}-{Z}_{2}+{R}_{s}}$$

where Z_1_, Z_2_ and R_s_ are impedance of electrodes 1, 2 and resistance of the solution, respectively. In many cases, especially in aqueous solutions, the R_s_ can be ignored. In such cases, Eq. (1) becomes the following equation^41^:

2 $$\Delta I=\frac{{Z}_{1}{i}_{1}-{Z}_{2 }{i}_{2}}{{Z}_{1}-{Z}_{2}}$$

These equations show that the current originated from the corrosion of one electrode is divided between the two WEs. Regarding corrosion events happening just on electrode 1 (i_2_ = 0) and generate a current (i_1_). Hence, it is clear from Eq. (2), for identical electrodes at the same potential (Z_1_ = Z_2_), only one-half of i_1_ flows to the other one while it can be recorded by the ZRA.

Conversely, if Z_2_ <  < Z_1_, approximately the whole of current transfer to the other electrode and measured by ZRA. Z_2_ <  < Z_1_ happens when the area of electrode 1 is much smaller than electrode 2. Impedance is inversely proportional to area^27^.

#### Statistical analysis

**Figure 2:**
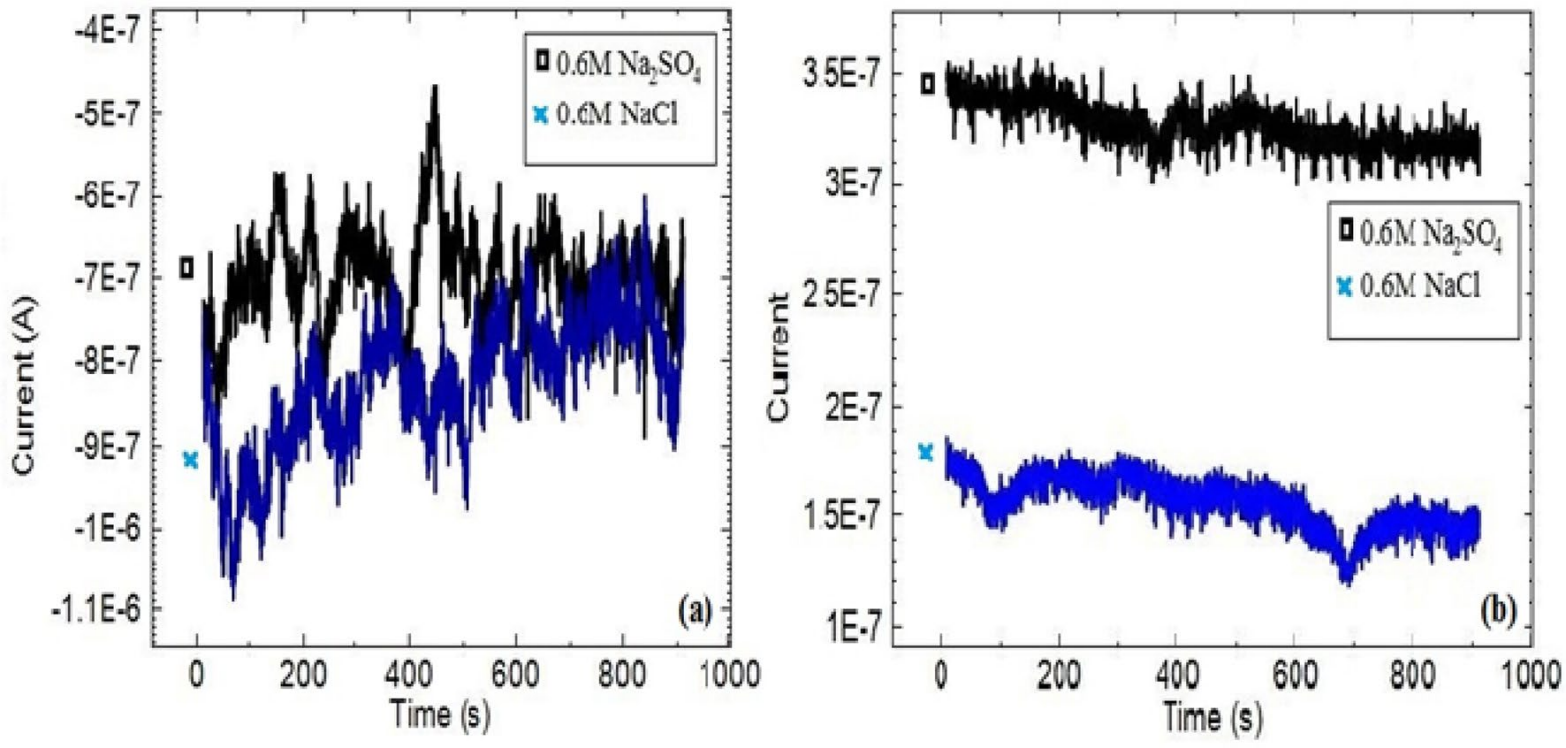
ECN signals of copper (**a**) Sym and (**b**) Asy electrodes in 0.6 M NaCl and 0.6 M Na_2_SO_4_ solutions at 60 °C.

**Figure 3:**
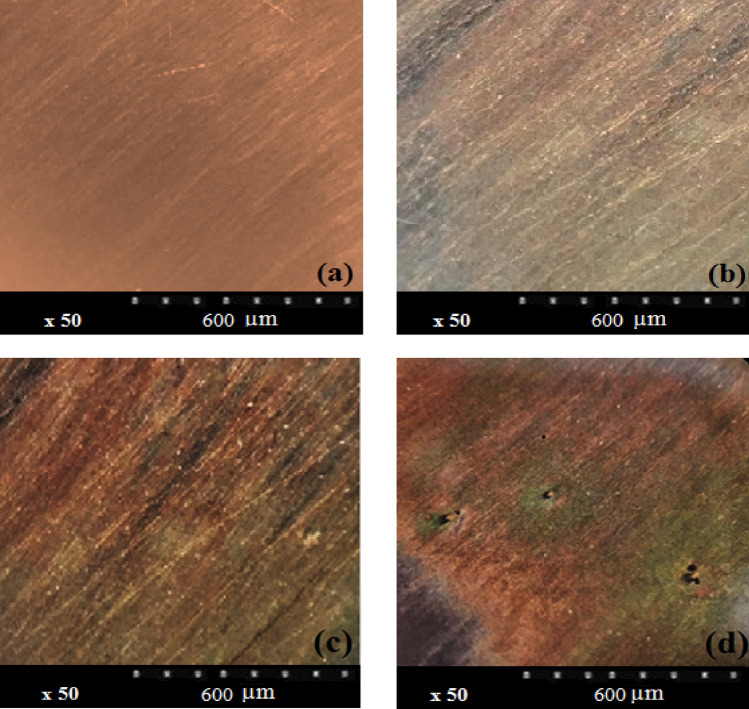
Optical micrographs of copper (**a**) before and (**b**–**d**) after immersion in 0.6 M NaCl, 0.6 M Na_2_SO_4_ and 0.3 M NaCl + 0.3 M Na_2_SO_4_ solutions at 60 °C.

Copper Sym and Asy electrodes were examined in each of 0.6 M NaCl and 0.6 M Na_2_SO_4_ solutions separately at 60 °C. No ECN signals that indicate pitting corrosion was recorded in the solution consisting of either Cl^−^ or SO_4_^2−^ ions (Fig. 2). Because any pitting corrosion has not occurred. Figure 3, optical micrographs clearly reveals the copper surface in varies solutions.

**Figure 4:**
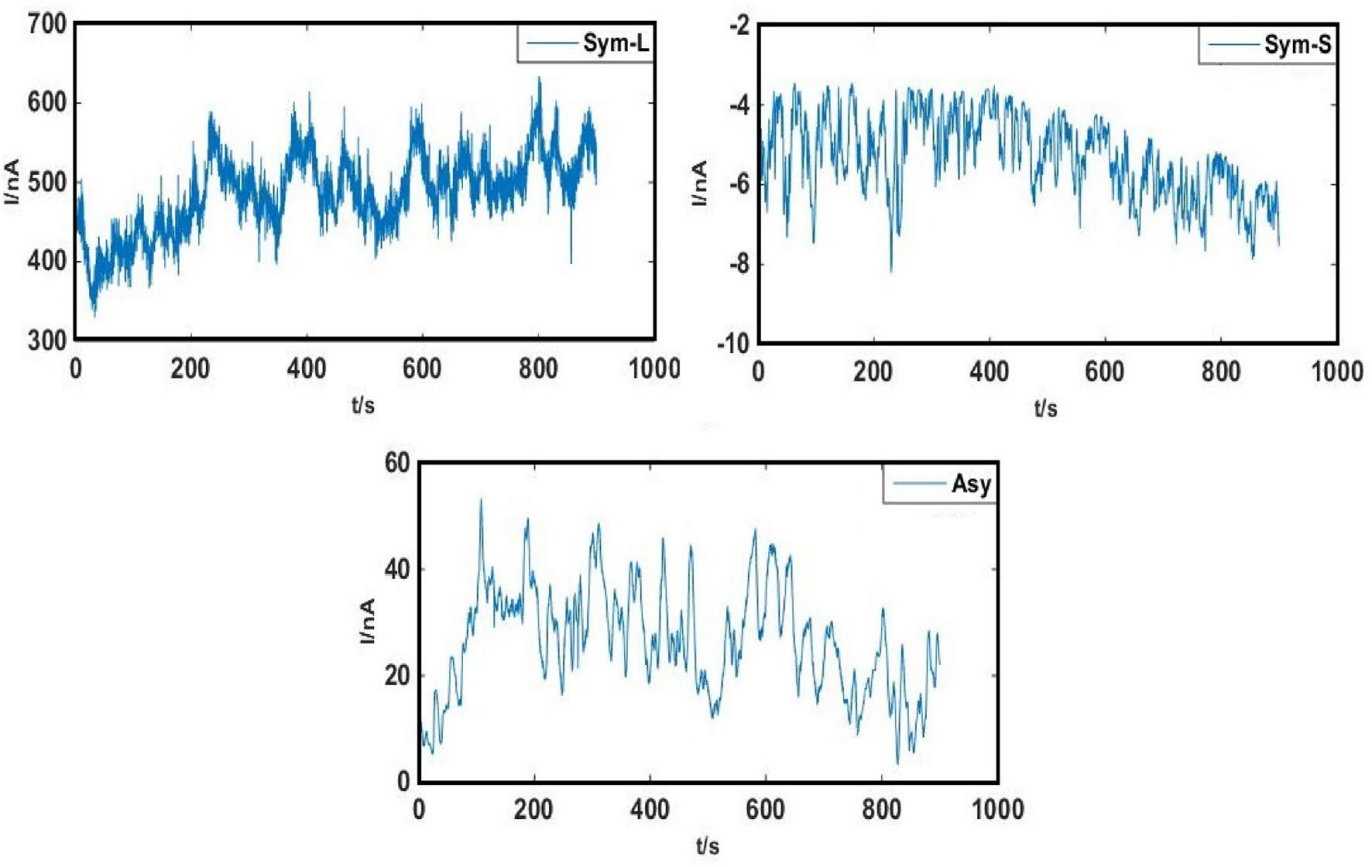
The original ECN signals of Sym and Asy electrodes in 0.3 M NaCl + 0.3 M Na_2_SO_4_ solution at 60 °C.

**Figure 5:**
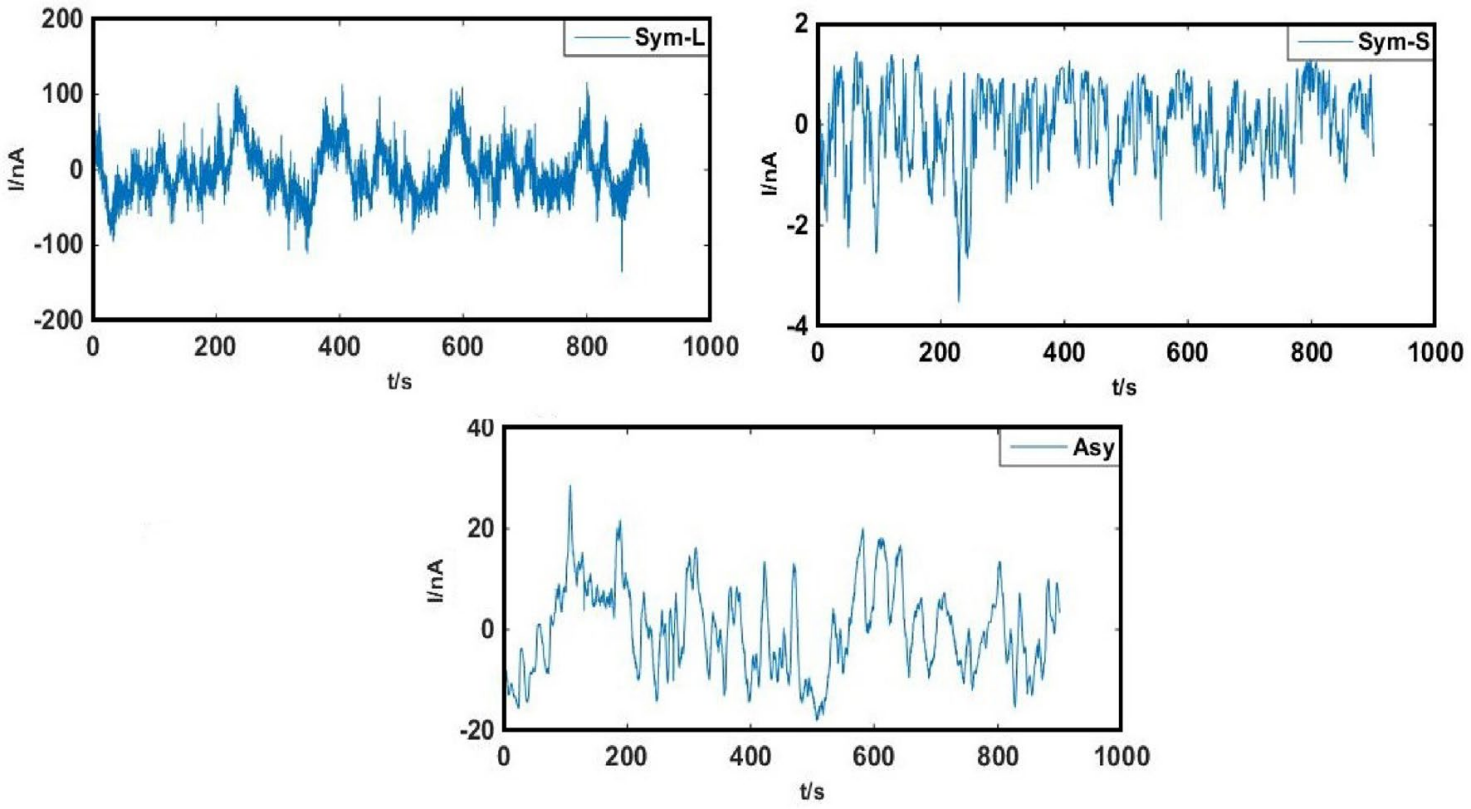
The ECN signals depicted in Fig. 4. After trend removal.

The signals were recorded using Sym and Asy copper electrodes in the mixture solution of 0.3 M NaCl + 0.3 M Na_2_SO_4_ for the pitting occurrence at 60 °C (pH 6). The WT procedure was used to detrend the raw electrochemical current noise transients (Fig. 4) with no changes in the practical data, the results of which are represented in Fig. 5.

Table 2 indicates the statistical parameters, higher values of standard deviation observed for signals of Sym-L compared to Sym-S electrodes (SD is a function of both the number and amplitude of the signals)^33^. The greater number (not the amplitude) of signals recorded by Sym-L electrodes can explain the SD differences due to lower kurtosis compared to signals recorded by Sym-S electrodes as shown in Fig. 5. In other words, the greater signal number leads to the lower values of kurtosis^20^. It is noteworthy that the use of Asy electrodes aims at avoiding the overlap of signals and improving the detection of noise through amplitude increase^33^. Hence, Sym-S electrodes are suitable candidates to be compared with Asy ones. As can be seen in Table 2, there is a considerably greater SD for the signals from Asy compared to Sym electrodes, potentially because of the greater noise signal amplitude in the Asy electrodes, due to their larger kurtosis, and consequently fewer noises. Table 2 and Fig. 5 show that Asy electrodes have high current signal skewness due to the unidirectionality of the signals, resulting from anodic and cathodic reactions occurring on the small and large electrodes, respectively. Considering insufficient space of the small electrode for reduction, the movement of the electrons produced from the anodic reactions takes place toward the larger electrode which is the place of cathodic reduction reactions^33^.

**Table 2:** Statistical parameters computed for current signals of Sym and Asy copper electrodes in 0.3 M NaCl + 0.3 M Na_2_SO_4_ at 60 °C.

Cells	SD	Skew	Kurt
Sym-L	42.1187	− 0.0594	0.0051
Sym-S	0.7277	− 0.0434	0.0656
Asy	6.2286	0.4252	0.2835

#### Wavelet analysis

**Figure 6:**
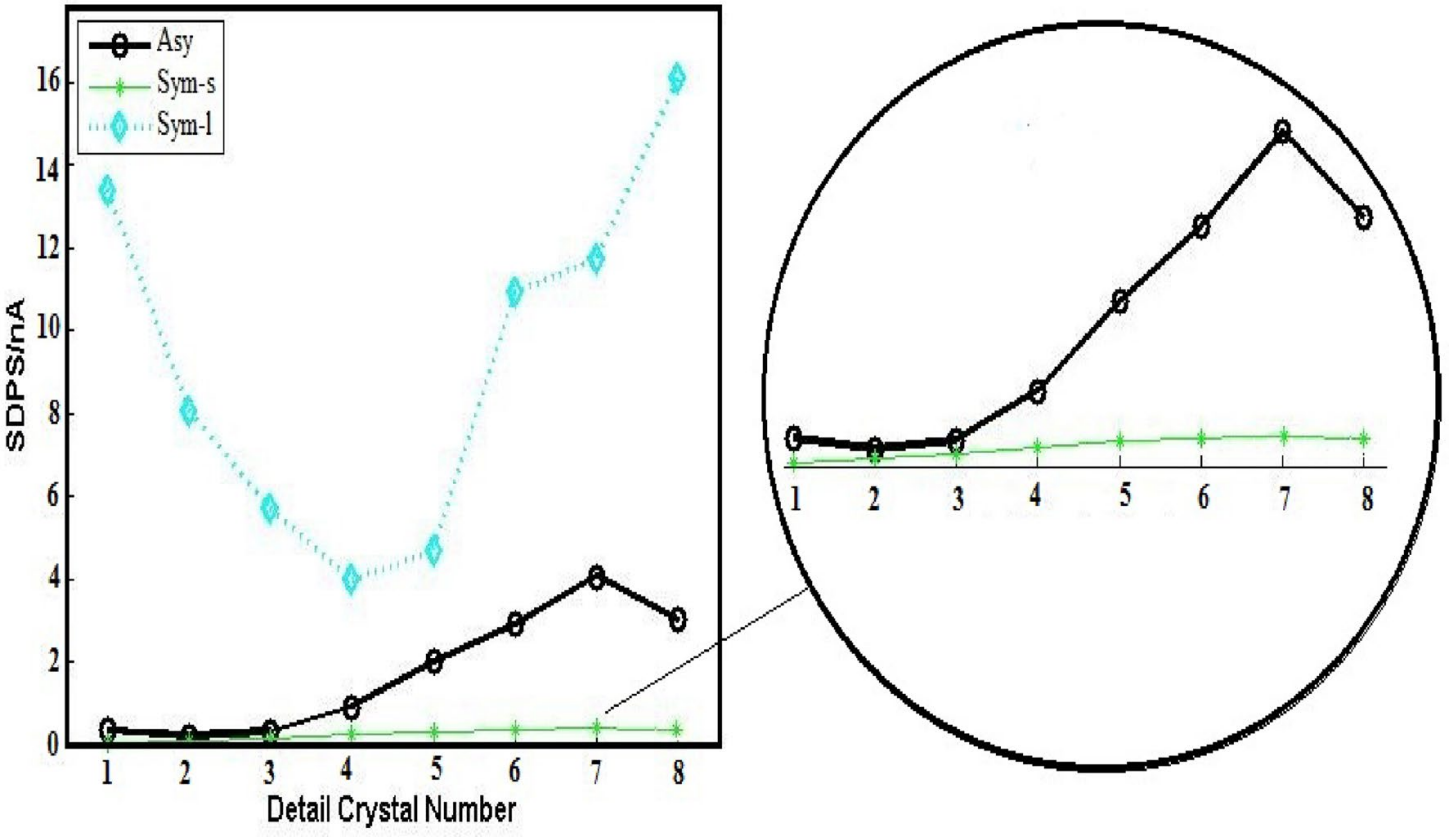
SDPS plots of Sym and Asy configuration ECN signals depicted in Fig. 5.

Figure 4 shows the decomposition of every set of 3600 data points of ECN signals taking place with the use of WT while the SDPS diagrams of the transients are represented in Fig. 6. The maximum peak position in the SDPS plots can be used for the detection of every predominant transient^35^.

**Figure 7:**
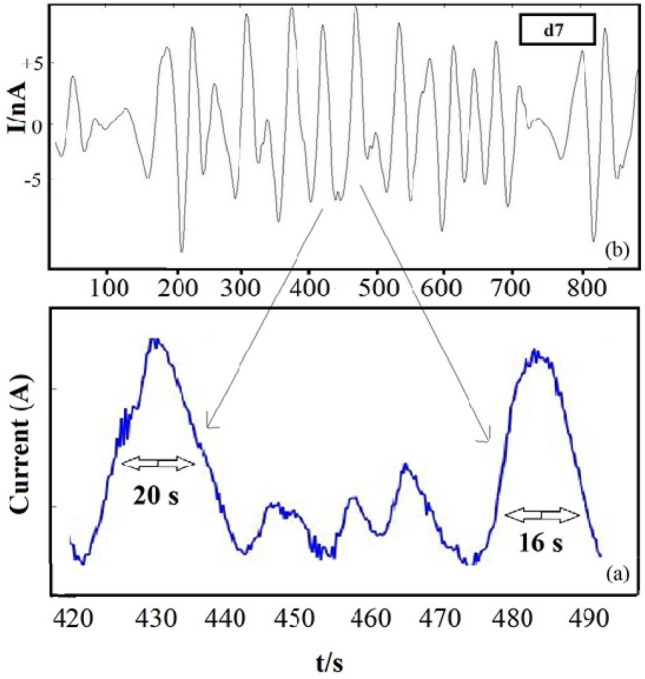
(**a**) The d7 partial signal, (**b**) part of the Asy original signal illustrated in Fig. 5.

The SDPS plot maximum peak location in Asy cells is d7 crystal which cannot be observed in the case of Sym-L and Sym-S (Fig. 6). The partial and original signals were compared to make sure about the accuracy of d7 crystal (transients with 16–32 s time scale as shown in Table 1). According to Fig. 7b, several peaks are found for the partial signal of d7 crystal in Asy electrodes at various times (such as 400 and 500 s). Based on optical micrographs in Fig. 3, these signals can lead to stable pits on the surface of copper. Figure 7a shows the original signals whose time scale is at a range of 16–32 s for each peak, allocated to crystal d7, without any overlaps. Thus, it needs to have trust in the observable maximum peak location of Asy electrodes, which means that there is no requirement to compare the partial and original signals, whereas such a comparison is necessary for Sym electrodes^37^.

**Figure 8:**
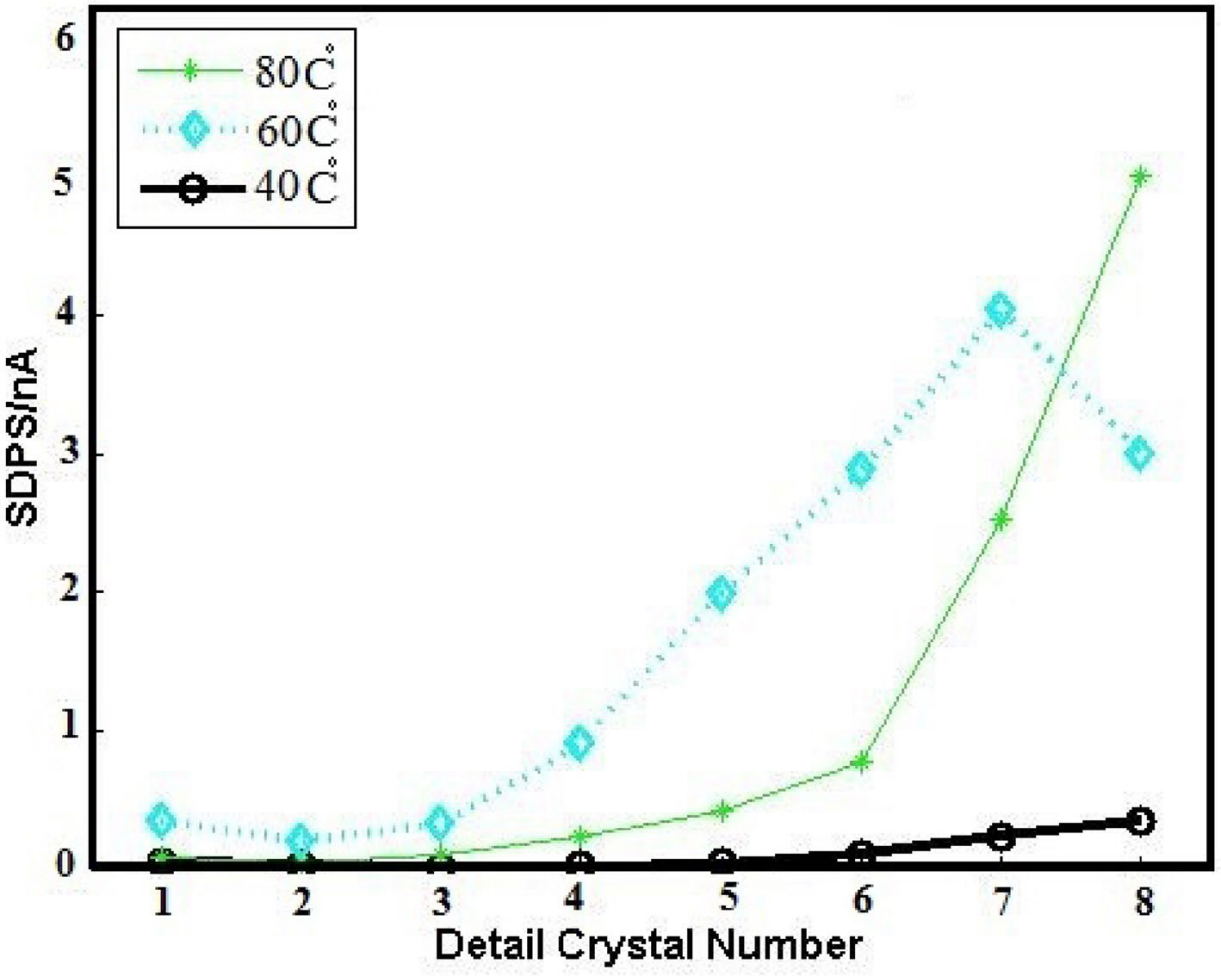
SDPS plots of Asy electrodes ECN signals in 0.3 M NaCl + 0.3 M Na_2_SO_4_ solution at 40, 60 and 80 °C.

Given the efficiency of Asy electrodes SDPS plots, temperatures of 40 and 80 °C are considered to examine the temperature impacts on copper corrosion by SDPS plots as can be seen in Fig. 8. The pit development leads to the flow of a quantity of electric charge (Q) in the circuit, whose estimation is carried out using the relation below^44^:

3 $$Q={SDPS}_{max}. {\tau }_{max}$$

**Figure 9:**
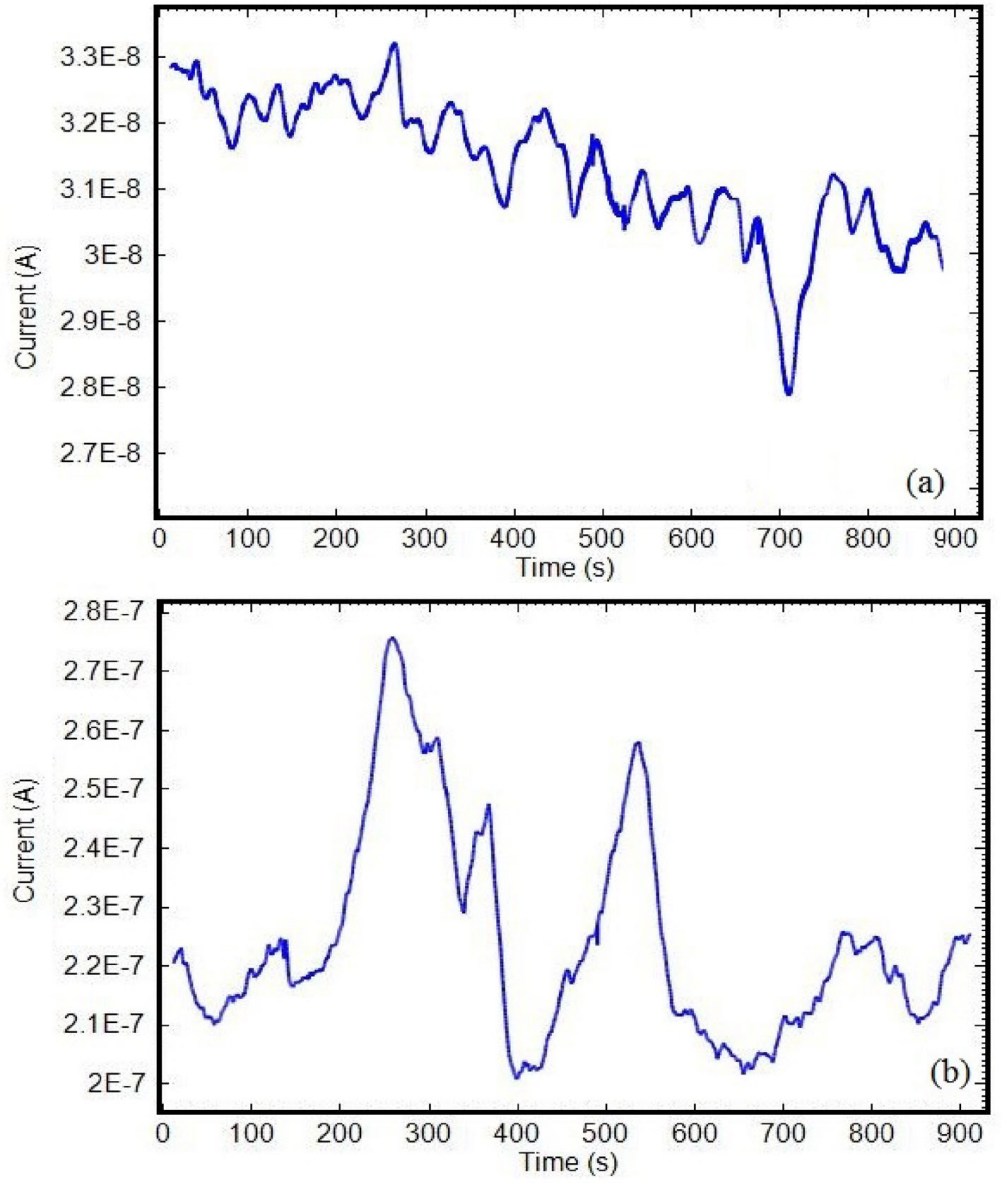
The original ECN signals of Asy electrodes in 0.3 M NaCl + 0.3 M Na_2_SO_4_ solution at (**a**) 40 °C and (**b**) 80 °C.

In which, SDPS_max_ and τ _max_ represent the values of the SDPS at the maximum peak crystal (d_max_) and the average time width of d_max_ crystal, respectively. Table 3 summarizes the parameter values taken from Fig. 8 and Table 1. Higher quantities of Q at 80 °C compared to the other temperatures are depicted, leading to more corrosion than the others. Also, the large signals at 80 °C compared to the small ones at 40 °C proved this claim (Fig. 9).

**Table 3:** The time scales of the predominant transients of SDPS plots corresponding to Asy copper electrodes in 0.3 M NaCl + 0.3 M Na_2_SO_4_ at 40, 60 and 80 °C.

T(°C )	SDPS_max_/nA	d_max_	τ_max_/S	Q/nCoul
40	0.025	d8	48	1.2
60	4.210	d7	24	101
80	5.33	d8	48	255

### EIS and polarization measurements

**Figure 10:**
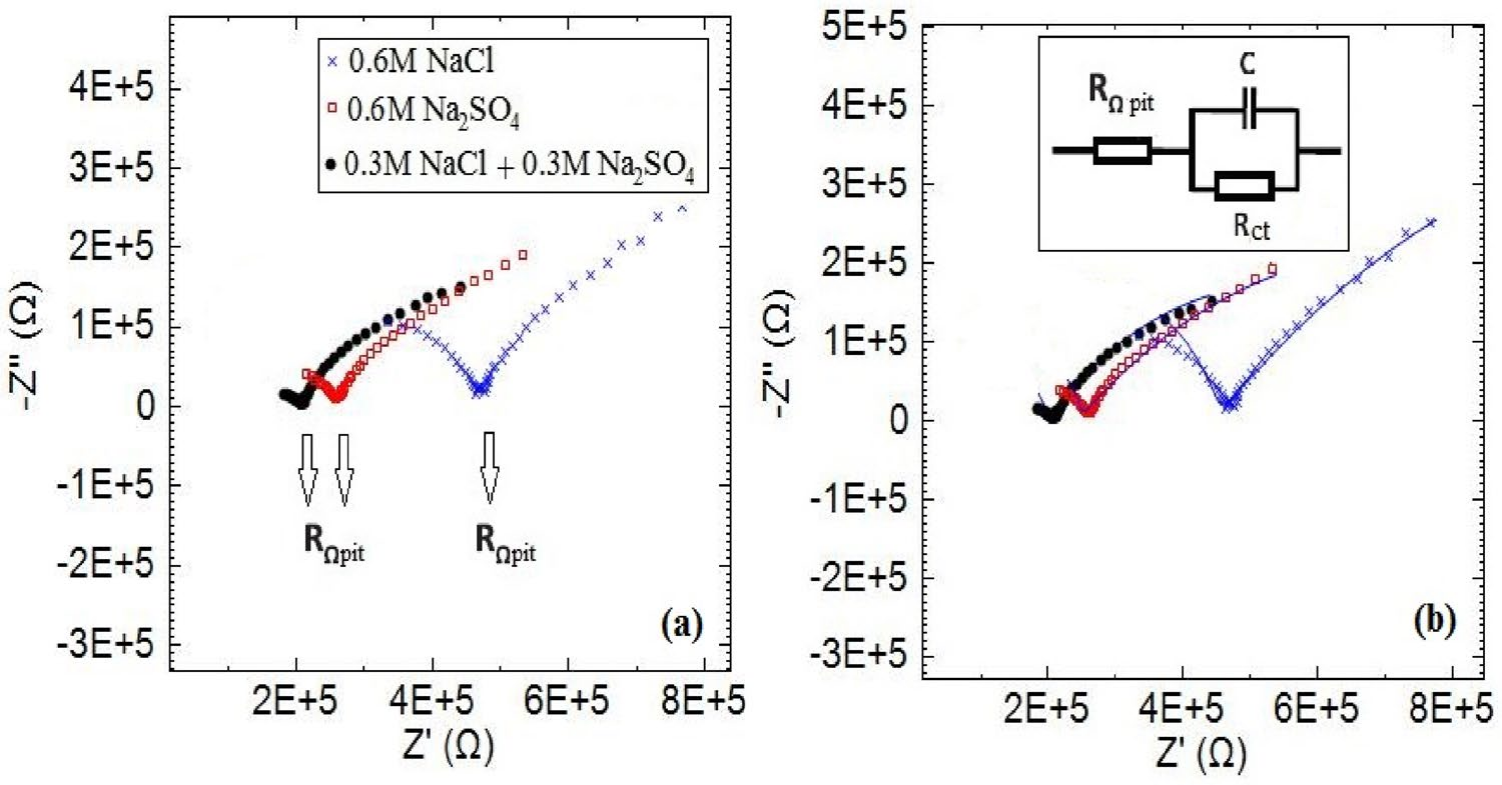
(**a**) The EIS plots of copper (3.14 mm^2^) in different solutions at 60 °C. (**b**) The acceptable fit of the experimental data and Randles circuit.

Following the results of the previous section, EIS and polarization measurements are performed to evaluate the pitting corrosion behavior of copper metal in the presence of chloride and sulfate ions. Most theories associated with copper pitting regarded chloride as accounting for pitting attacks while excluding the direct contribute on of sulfate ions^38,45,46^. According to the previous section and the other research, in the presence of both sulfate and chloride ions, as an aggressive medium, the pit germination is mainly associated with the presence of both these species^45,46^. The normal EIS measurements conducted in 0.3 M NaCl + 0.3 M Na_2_SO_4_ 0.6 M NaCl and 0.6 M Na_2_SO_4_ solutions at 60 °C can be observed in Fig. 10. The sum of the charge transfer resistance and an ohmic resistance reflects at the low frequencies region in the Nyquist plot. The tail at high frequencies is allocated to an ohmic resistance shown using the arrow as R_Ωpit_ of the Nyquist plot in Fig. 10a. This Ohmic resistance was verified by spiking solutions containing pit free copper electrodes in the same geometry as artificial pits with supporting electrolyte to shift the point indicated as R_Ωpit_^38^. According to Fig. 10b, the semi-circle in the Nyquist plot was fitted to a Randles circuit to obtain the pit ohmic resistance and charge transfer values. The best fit plots and Randles circuit as shown in Fig. 10b.

Referring to Table 4, the ohmic and charge transfer resistances in the solution of 0.3 M NaCl + 0.3 M Na_2_SO_4_ were less than those obtained from solutions having either chloride or sulfate. Even though there are different opinions about the corrosion mechanism of copper in chloride solution, it is generally accepted that insoluble salt film CuCl is formed; however, this kind of salt film cannot be formed in the sulfate solution, as a result, the pitting corrosion is enhanced in the mixture solution of Cl^−^ and SO_4_^2−^ ions^45,46^.

**Table 4:** The EIS parameters of copper electrode (3.14mm^2^) at 60 °C.

Solutions	R_ct_ (kΩ)	R_pit_ (kΩ)
NaCl	1200	470
Na_2_SO_4_	1000	260
NaCl + Na_2_SO_4_	900	210

**Figure 11:**
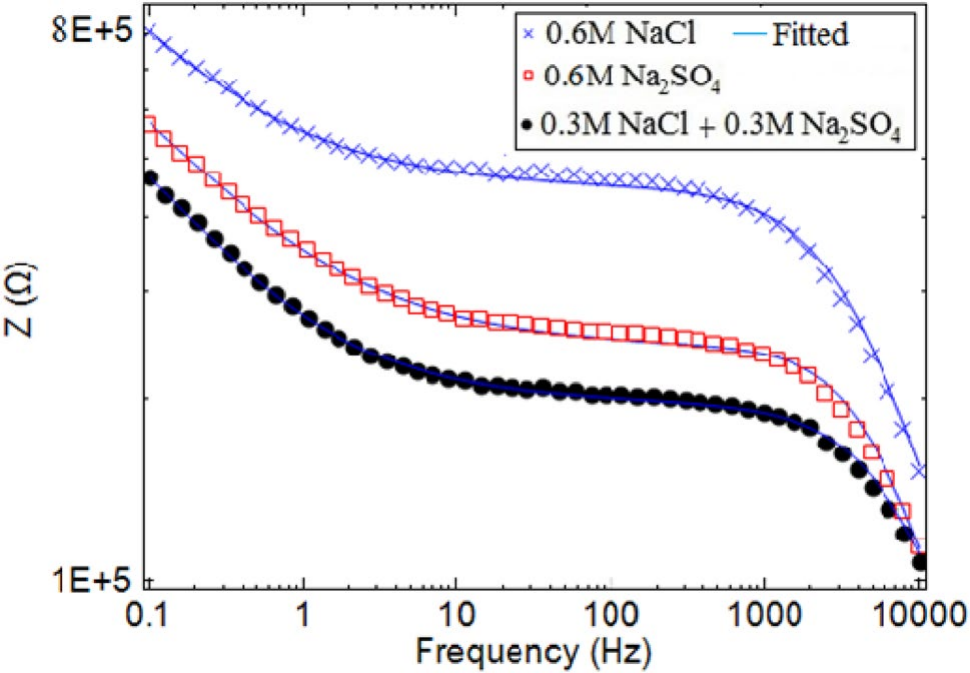
The Bode plots of copper (3.14 mm^2^) in different solutions at 60 °C.

Besides, the evaluation of the passive oxide layer thickness is possible by using bode diagrams (thicker oxide films reflected by greater slopes and y-intercepts)^37^. It is clear from Fig. 11, the oxide layer is less thick on the copper when using 0.3 M NaCl + 0.3 M Na_2_SO_4_ compared to the other solutions. Accordingly, the oxide layer had sufficiently low resistance to attack the ions to the copper surface from the solution containing both chloride and sulfate and there is a synergistic effect of chloride and sulfate ions on copper pitting corrosion.

**Figure 12:**
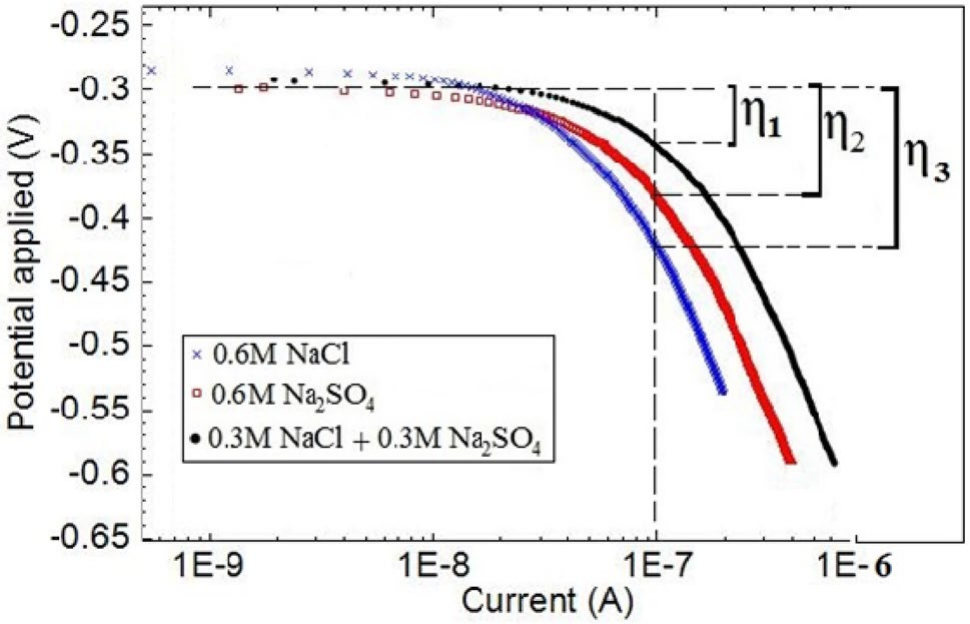
The polarization plots of copper (3.14 mm^2^) in different solutions at 60 °C.

The polarization plots for corrosion of copper in different media are depicted in Fig. 12. Over potential (η) values, which are a measure of the rate of reduction reaction on the surface electrode, are calculated using the following equation^42^:

4 $$\eta ={E}_{appl}-{E}_{eq}$$

where E_appl_ and E_eq_ represent the values of applied potential (to obtain constant current) and equilibrium potential, respectively. In other word, the over potential is the potential of cell relative to its equilibrium value. Here, considerably less over potential values are obtained for the solution containing chloride + sulfate (η_1_) compared to the solutions of Na_2_SO_4_ (η_2_) and NaCl (η_3_) in Table 5. It shows that there is a sufficiently high rate of oxygen reduction (subsequently high rate of anodic reaction) on the less thick of copper oxide layer in the mixture solution for pitting occurrence. The more negativity of the corrosion potential which leads to pitting corrosion results from the highest cathodic reaction rate in this situation^43^.

**Table 5:** The over potential values of copper electrode (3.14mm^2^) at 60 °C.

Solutions	E_appl_ (V)	E_eq_ (V)	η (V)
NaCl	− 0.457	− 0.300	− 0.157
Na_2_SO_4_	− 0.410	− 0.300	− 0.110
NaCl + Na_2_SO_4_	− 0.351	− 0.300	− 0.051

### DFT calculation

As mentioned above, Cl^−^ and SO_4_^2−^ ions facilitate the corrosion process of pure copper metal. Once all the parameters are the same in experiments, the effects of Cl^−^ and SO_4_^2−^ on the corrosion process of Cu metal could be attributed to the stabilization of Cu^2+^ cations by Cl^−^ and SO_4_^2−^ ions in the aqueous solutions. Here, the reactions of Cl^−^ and SO_4_^2−^ ions with Cu^2+^ cations in aqueous media are modeled. In addition, it is attempted to properly account for the effects of water molecules on the stabilization processes. The interactions of H_2_O molecules with ions of the system could be divided into explicit interactions and implicit interactions^47–49^. In order to account for the explicit interactions of H_2_O molecules with ions, Cu^2+^ cation is considered as a complex with six coordinated water molecules and Cl^−^ and SO_4_^2−^ ions as the anions, hydrated with three and four water molecules, respectively. Next, it was supposed that the stabilization of Cu^2+^ cations by Cl^−^ and SO_4_^2−^ ions occurred according to the following processes:

$${\left[Cu{\left({H}_{2}O\right)}_{6}\right]}^{2+}+4 {Cl}^{-}{\left({H}_{2}O\right)}_{3} \to {\left[Cu{\left(Cl\right)}_{4}\right]}^{2-}{\left({H}_{2}O\right)}_{18} \qquad R1$$

$${\left[Cu{\left({H}_{2}O\right)}_{6}\right]}^{2+}+2 S{O}_{4}^{2-}{\left({H}_{2}O\right)}_{4}\to {\left[Cu{\left(S{O}_{4}\right)}_{2}\right]}^{2-}{\left({H}_{2}O\right)}_{14} \qquad R2$$

$${\left[Cu{\left({H}_{2}O\right)}_{6}\right]}^{2+}+2 {Cl}^{-}{\left({H}_{2}O\right)}_{3}+ S{O}_{4}^{2-}{\left({H}_{2}O\right)}_{4}\to {\left[Cu{\left(Cl\right)}_{2}S{O}_{4}\right]}^{2-}{\left({H}_{2}O\right)}_{16} \qquad R3$$

**Figure 13:**
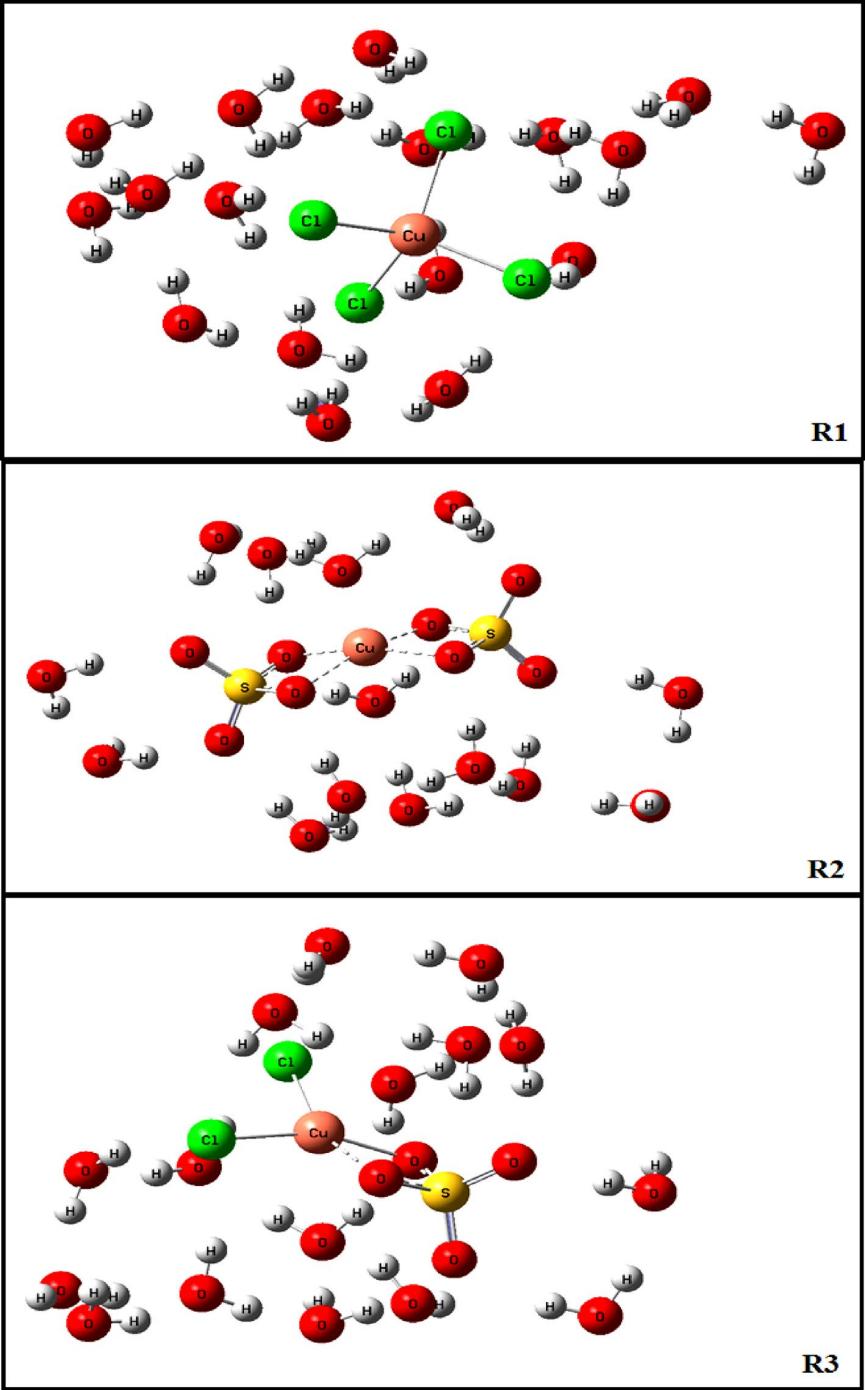
The structures of the species involved in the reactions R1, R2 and R3.

The structures of the species involved in the reactions R1, R2 and R3 are depicted in Fig. 13. The computed reaction energies for R1, R2 and R3 are − 347.1 kJ/mol, − 351.5 kJ/mol and − 373.5 kJ/mol, respectively. These computed energies are in accordance with the trend observed for the effects of Cl^−^ and SO_4_^2−^ anions on corrosion acceleration of copper metal. The reaction of Cu^2+^ cation with SO_4_^2−^ anions (the reaction R2) leads to a more stable complex than that with Cl^−^ anions (the reaction R1). A mixture of Cl^−^ and SO_4_^2−^ anions stabilize Cu^2+^ cations more than individual Cl^−^ or SO_4_^2−^ anions (reaction R3). The present DFT calculations provide an explanation for the synergistic effects of Cl^−^ or SO_4_^2−^ anions in increasing the corrosion rate of copper metal.

## Conclusions

In the present study, the corrosion behavior of pure copper was investigated by using ECN, EIS and polarization methods in the solution consisting of Cl^−^ and SO_4_^2−^ as aggressive ions. A key objective of this paper has been to reveal the benefits of using Asy electrodes in electrochemical noise measurements for copper pitting corrosion. The conclusions can be drawn as follows:

1. The electrodes surface area of the Asy configuration (3–300 mm^2^) demonstrated an appreciable enhancement detection of pitting corrosion.
2. The normal justification for using Asy configuration is to allow the study of what is happening on one of the two electrodes. That here statistical parameters of the Asy configuration indicated recorded data are related to the small electrode. However, the large electrode is the substrate that consumed the produced electrons.
3. The SDPS plots of Asy copper configuration are more efficient than those of Sym one because the apparent maximum signal location arising from the Asy electrodes is actual and there is no requirement to compare the partial and original signals, whereas such a comparison is necessary for Sym electrodes.
4. The SDPS plots illustrated that corrosion increased on copper with increasing temperature.
  Because of higher quantities of Q at 80 °C compared to the other temperatures.
5. The ECN technique examined pitting corrosion in the mixture solution because salt film (CuCl) cannot be formed in the sulfate solution, as a result, the pitting corrosion is enhanced due to synergetic effects of chloride and sulfate ions and showed no signals related to pitting corrosion in each of NaCl and Na_2_SO_4_ solutions.
6. Nyquist and bode plots indicated that corrosion was increased in the presence of both Cl^−^ and SO_4_^2−^ ions which the oxide layer resistance was sufficiently low to get the ions on the copper surface. It was consistent with the result obtained from ECN as well as optical micrographs.
7. Polarization method is efficient method to detect corrosion by measuring of cathodic reaction rate. The highest cathodic reaction rate in the presence of both the ions leads to the pitting corrosion resulting from the higher negativity of the corrosion potential.
8. The DFT calculations provided an explanation for the stabilization of Cu^2+^ cations by synergistic effect of Cl^−^ or SO_4_^2−^ ions in aqueous solutions. The calculated results were consistent with the experimental data.

## Data Availability

The datasets used and/or analysed during the current study available from the corresponding author on reasonable request.
